# A Novel Zinc Finger Protein Zfp277 Mediates Transcriptional Repression of the *Ink4a/Arf* Locus through Polycomb Repressive Complex 1

**DOI:** 10.1371/journal.pone.0012373

**Published:** 2010-08-24

**Authors:** Masamitsu Negishi, Atsunori Saraya, Shinobu Mochizuki, Kristian Helin, Haruhiko Koseki, Atsushi Iwama

**Affiliations:** 1 Department of Cellular and Molecular Medicine, Graduate School of Medicine, Chiba University, Chiba, Japan; 2 RIKEN Research Center for Allergy and Immunology, Laboratory for Developmental Genetics, Yokohama, Japan; 3 Biotech Research and Innovation Centre (BRIC) and Centre for Epigenetics, University of Copenhagen, Copenhagen, Denmark; 4 JST, CREST, Tokyo, Japan; Centre National de la Recherche Scientifique, France

## Abstract

**Background:**

Polycomb group (PcG) proteins play a crucial role in cellular senescence as key transcriptional regulators of the *Ink4a/Arf* tumor suppressor gene locus. However, how PcG complexes target and contribute to stable gene silencing of the *Ink4a/Arf* locus remains little understood.

**Methodology/Principal Findings:**

We examined the function of Zinc finger domain-containing protein 277 (Zfp277), a novel zinc finger protein that interacts with the PcG protein Bmi1. Zfp277 binds to the *Ink4a/Arf* locus in a Bmi1-independent manner and interacts with polycomb repressor complex (PRC) 1 through direct interaction with Bmi1. Loss of *Zfp277* in mouse embryonic fibroblasts (MEFs) caused dissociation of PcG proteins from the *Ink4a/Arf* locus, resulting in premature senescence associated with derepressed *p16^Ink4a^* and *p19^Arf^* expression. Levels of both Zfp277 and PcG proteins inversely correlated with those of reactive oxygen species (ROS) in senescing MEFs, but the treatment of *Zfp277* ^−/−^ MEFs with an antioxidant restored the binding of PRC2 but not PRC1 to the *Ink4a/Arf* locus. Notably, forced expression of *Bmi1* in *Zfp277* ^−/−^ MEFs did not restore the binding of Bmi1 to the *Ink4a/Arf* locus and failed to bypass cellular senescence. A *Zfp277* mutant that could not bind Bmi1 did not rescue *Zfp277* ^−/−^ MEFs from premature senescence.

**Conclusions/Significance:**

Our findings implicate Zfp277 in the transcriptional regulation of the *Ink4a/Arf* locus and suggest that the interaction of Zfp277 with Bmi1 is essential for the recruitment of PRC1 to the *Ink4a/Arf* locus. Our findings also highlight dynamic regulation of both Zfp277 and PcG proteins by the oxidative stress pathways.

## Introduction

Cellular senescence is a fundamental cellular program triggered after a finite number of cell divisions (replicative senescence) or more rapidly in response to acute stress (premature senescence). It functions as a cell-autonomous safeguard against severe genomic instability and carcinogenesis and contributes to aging in mammals as well. This irreversible cytostatic state can be triggered by multiple mechanisms, including telomere shortening, oxidative stress, cancer-causing genetic alterations, and DNA damage [1], [2]. These intrinsic and extrinsic stress factors subsequently induce epigenetic derepression of the *Ink4a/Arf* tumor suppressor gene locus.

The *Ink4A/Arf* or *Cdkn2a* locus contains two distinct tumor suppressors, *p16^Ink4^* and *p19^Arf^* (*p14^ARF^*in humans) [3], [4]. p16^Ink4a^ protein directly inhibits the activities of the cyclin D-dependent kinases, Cdk4 and Cdk6, and maintains the pRb-E2f pathway in an anti-proliferative state, while p19^Arf^ controls the stabilization and activation of p53 through binding to Mdm2, and promotes cellular senescence in primary fibroblasts [5]. Although both p16^Ink4a^ and p19^Arf^ play an important role in cellular mortality [6], [7], [8], there seem to be species-specific differences in their function. For example, the p16^INK4A^-pRB-E2F pathway plays a major role in regulating the cellular senescence in human primary fibroblasts, whereas the p19^Arf^-Mdm2-p53 pathway does so in MEFs [9], [10].

PcG complexes are key regulators of epigenetic cellular memory. They establish and maintain cellular identities during embryogenesis, development, and tumorigenesis [11]. They have also been implicated in the maintenance of embryonic and somatic stem cells [12]. Among PcG proteins, Bmi1 and its role in the inheritance of the stemness of adult somatic stem cells have been well characterized [13], [14]. One of the major targets of the PcGs, including Bmi1, is the *Ink4a/Arf* locus. Loss of *Bmi1* causes derepression of the *Ink4a* and *Arf* genes, resulting in premature fibroblast senescence and depletion of hematopoietic and neural stem cells [13], [14], [15]. Conversely, forced expression of *Bmi1* extends the replicative life span of MEFs in tissue culture and enhances stem cell activity, at least partially, by repressing the *Ink4a/Arf* locus [16], [17], [18], [19]. These observations indicate that PcG proteins are critical regulators of cellular senescence through the *Ink4a/Arf* locus.

Biochemical and genetic studies have identified that PcG complexes can be functionally separated into at least two distinct complexes: an initiation complex, PRC2, and a maintenance complex, PRC1. The human PRC2 core complex contains EZH2, EED, SUZ12, and RBAP48 (Nurf55 in *Drosophila*). PRC2 possesses catalytic activity specific for the trimethylation of histone H3 at lysine 27 (H3K27me3) and mono- and dimethylation of histone H1 at lysine 26 (H1K26me1/me2) [11], [20], [21]. The trimethylation of H3K27 is a repressive histone modification essential for gene silencing by PcGs and it has been suggested that this epigenetic mark functions to recruit PRC1 to chromatin [22]. By contrast, the PRC1 core complex contains RING2 (Ring1B in mice), BMI1, HPH, and CBX and possesses E3 ubiquitin ligase activity for the monoubiquitination of histone H2A at lysine 119 (H2AK119ub1) [21], [23]. H2AK119ub1 has been suggested to play a central role in PRC1-mediated gene repression [24], [25] and some data indicate that this modification interferes with translational elongation by restraining poised RNA polymerase II at bivalent genes in embryonic stem (ES) cells [26].

To understand the molecular mechanism of PcG gene silencing, we previously performed a yeast two-hybrid screening and identified a novel Bmi1-interacting protein, Zpf277 (known as ZNF277 in humans). In this study, we show that Zfp277 is required for the stable accumulation of PRC1 at the *Ink4a/Arf* locus and plays a critical role in the regulation of cellular senescence.

## Results

### Zfp277 binds to Bmi1 in mammalian cells

In a previously published yeast two-hybrid screening [27], we identified Zpf277 as a Bmi1-binding protein (data not shown). Mouse Zfp277 showed greater than 80% identity to human ZNF277 at the amino acid level and shared five highly conserved C_2_H_2_ zinc finger motifs (Figure. S1A). Human *ZNF277* was first documented as a gene located on chromosome 7 at q31 that is often deleted in several malignancies and autism [28], [29]. Both mouse *Zfp277* and human *ZNF277* are ubiquitously expressed in a variety of tissues, including brain, heart, spleen, lung, and liver [28] (data not shown). However, the function of Zfp277 has not yet been described.

**Figure 1:**
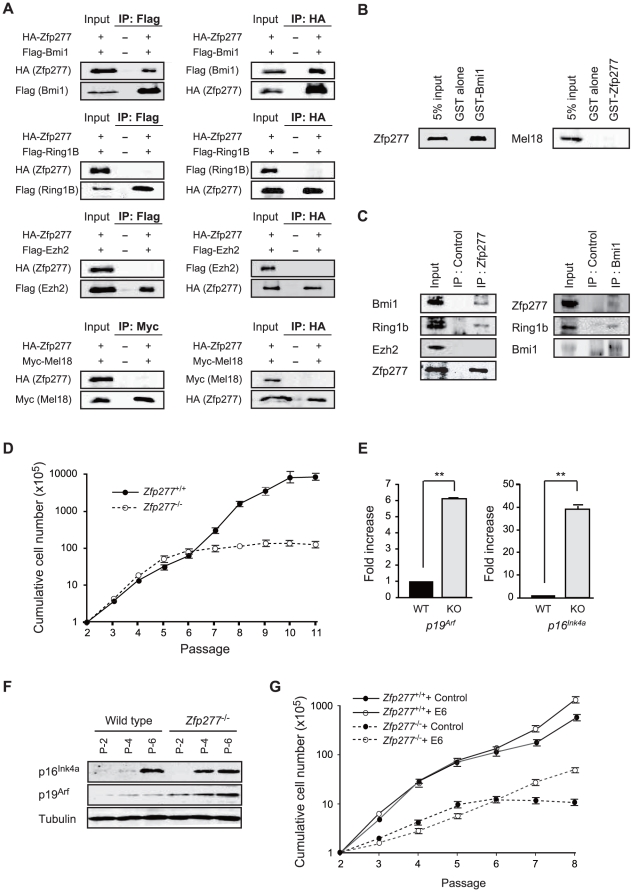
Zfp277 interacts physically with Bmi1. **A.** Zfp277 interacts with Bmi1 *in vivo*. HA-Zfp277 and Flag-tagged Bmi1, Ring1B, Ezh2, and Myc-tagged Mel18 were cotransfected into human 293T cells and the immunoprecipitates were subjected to a Western blot analysis using the antibodies indicated on the left. **B.** GST-pulldown between GST-Bmi1 and *in vitro*-translated Zfp277 (left panel) and GST-Zfp277 and *in vitro*-translated Mel18 (right panel). **C.** Binding of Zfp277 with PRC1 in MEFs. The whole-cell extracts from MEFs were immunoprecipitated with rabbit anti-Zfp277 serum (left panel) and anti-Bmi1 monoclonal antibody (right panel) or control IgG (control) and then subjected to Western blotting using the antibodies indicated on the left. A 2% input was loaded on the left. **D.** The growth curve of wild-type and *Zfp277* ^−/−^ MEFs. MEFs were seeded at 1×10^5^ cells/well in 6-cm plates and replated at 1×10^5^ cells/well every three days. Cumulative cell numbers are shown as the mean ± SD for three independent triplicate experiments. **E.** Quantitative RT-PCR analysis of *p19^Arf^* and *p16^Ink4a^* mRNA in MEFs at passage 2. mRNA levels were normalized to *Hprt1* expression. Expression levels relative to those in the wild-type MEFs are shown as the mean ± SD for three independent experiments. **F.** Expression of p19^Arf^ and p16^Ink4a^ in *Zfp277* ^−/−^ MEFs. The protein levels of p19^Arf^ and p16^Ink4a^ in wild-type and *Zfp277* ^−/−^ MEFs at the indicated passages were determined by Western blot analyses. Tubulin was used as a loading control. **G.** Rescue of the growth of *Zfp277* ^−/−^ MEFs by E6. Wild-type and *Zfp277* ^−/−^ MEFs were transduced with either a control or an *E6* retrovirus and cell growth was monitored every three days by replating at 1×10^5^ cells/plate. Cumulative cell numbers are shown as the mean ± SD for three independent triplicate experiments. Statistical significance was determined with Student's t-test; **p<0.01.

To confirm the physical interaction between Zfp277 and PcG proteins in mammalian cells, we conducted co-immunoprecipitation analyses of HA-tagged Zfp277 and Flag-tagged Bmi1, Ring1B, Ezh2, and Myc-tagged Mel18 using human 293T cells. As shown in Figure 1A, Zfp277 co-immunoprecipitated with Bmi1, but not with Ring1B or Ezh2. Of interest, Zfp277 did not co-immunoprecipitate with Mel18, another *Drosophila* Psc paralog in mammals which is 70% identical to Bmi1 at the amino acid level (Figure 1A). Next we employed a GST *in vitro* binding assay to confirm the physical interaction between Zfp277 and Bmi1 or Mel18 using GST-Bmi1 and GST-Zfp277, respectively. We confirmed that Zfp277 bound to Bmi1, but not to Mel18 (Figure 1B). Furthermore, endogenous PRC1 proteins, including Bmi1 and Ring1B, but not the PRC2 protein, Ezh2, were co-immunoprecipitated with Zfp277 using an anti-Zfp277 polyclonal antibody from the whole-cell lysate of MEFs (Figure 1C). Conversely, Zfp277 and Ring1B were co-immunoprecipitated with Bmi1 using an anti-Bmi1 antibody (Figure 1C). These findings suggested that Zfp277 interacts with PRC1 through direct binding to Bmi1, but not with PRC2.

### *Zfp277* ^−/−^ MEFs undergo premature senescence

To explore the function of *Zfp277*, we generated *Zfp277* knockout mice (Figure S1B). Exon 5 and its flanking regions were replaced with a neomycin-resistance gene cassette by homologous recombination in ES cells. The presence of the *Zfp277* knockout allele was confirmed by Southern blot analysis (Figure S1C). Western blot analysis using an anti-Zfp277 polyclonal antibody also confirmed the absence of Zfp277 protein in *Zfp277* ^−/−^ MEFs (Figure S1D).

*Zfp277* ^−/−^ mice were born healthy and fertile (data not shown). Although detailed analyses of their phenotypes are underway, we found that *Zfp277* ^−/−^ MEFs from E12.5 embryos undergo premature senescence at early passages (Figure 1D). *Zfp277* ^−/−^ MEFs at passage 6 exhibited a flattened and enlarged appearance and were highly positive for senescence-associated β-galactosidase activity, a marker for cellular senescence (Figure S2). Expression of p16^Ink4a^ and p19^Arf^ was highly up-regulated in *Zfp277* ^−/−^ MEFs compared to their wild-type counterparts at early passages at both the mRNA and protein levels (Figure 1E and F). p19^Arf^ reportedly plays a principal role in cellular senescence in mouse primary fibroblasts [9]. Correspondingly, premature senescence of *Zfp277* ^−/−^ MEFs was canceled by human papilloma virus-16 E6, which blocks the p19-Mdm2-p53 pathway [6] (Figure 1G), indicating that loss of *Zfp277* results in premature senescence of MEFs via the activation of the *Ink4a/Arf* locus.

### Zfp277 binds to the *Ink4a/Arf* locus

**Figure 2:**
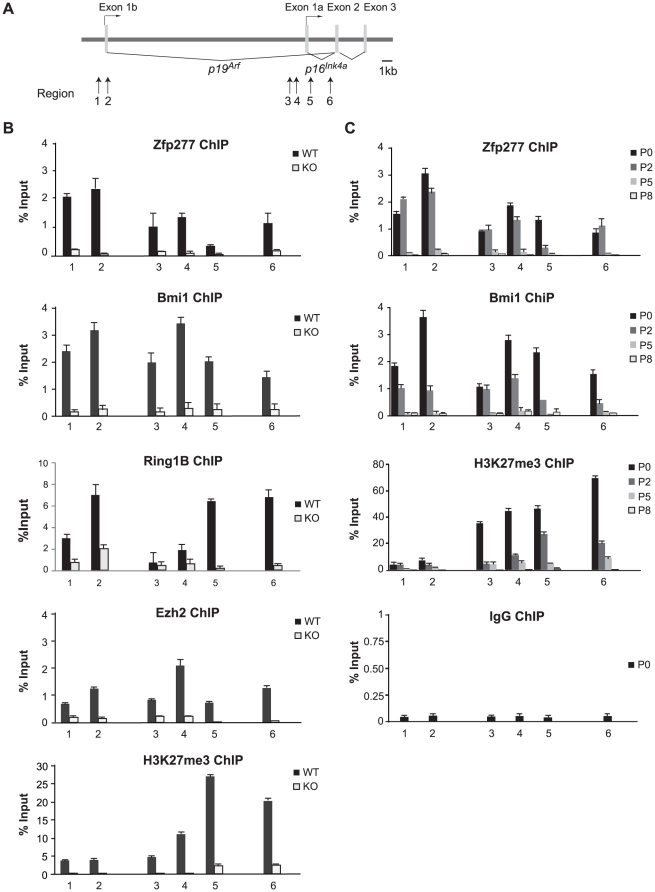
Zfp277 binds the *Ink4a/Arf* locus and is downregulated during serial passaging. **A.** A schematic representation of the *Ink4a/Arf* locus. ChIP assays were performed using anti-Zfp277, Bmi1, Ring1B, Ezh2, and H3K27me3 antibodies. Regions amplified from the precipitated DNA by site-specific quantitative PCR are indicated by arrows. **B.** Q-ChIP analysis of the *Ink4a/Arf* locus in wild-type or *Zfp277* ^−/−^ MEFs at passage 2 and (C) in wild-type MEFs at the indicated passages. Percentages of input DNA are shown as the mean ± S.D. for three independent experiments.

Recent studies revealed that PcG proteins are downregulated and dissociate from the *Ink4a/Arf* locus when cells are exposed to intra- or extracellular stress, including tissue culture- and oncogene-induced stress [30]–[33]. To understand the functional crosstalk between Zfp277 and PcGs, we next tested if Zfp277 is associated with the *Ink4a/Arf* locus. Quantitative chromatin immunoprecipitation (Q-ChIP) assays were performed using antibodies specific for Zfp277, PcG proteins (Bmi1, Ring1B, Ezh2), and PcG histone modifications (H2AK119ub1 and H3K27me3) over the *Ink4a/Arf* locus in MEFs (Figure 2A and B). As demonstrated in Fig. 2B, Zfp277 as well as the PcG proteins and their histone modifications were broadly distributed across the *Ink4a/Arf* locus in presenescent MEFs. Notably, Zfp277 was dissociated from the *Ink4a/Arf* locus during serial passages like the PcG proteins and H3K27me3 (Figure 2C). In addition, the binding of PcG proteins and levels of H3K27 trimethylation exhibited a significant reduction throughout the *Ink4a/Arf* locus in *Zfp277* ^−/−^ MEFs to levels comparable to those in the wild-type MEFs at late passages (Figure 2B and C). These findings suggested that Zfp277 participates in the PcG-mediated gene silencing of the *Ink4a/Arf* locus.

### Levels of Zfp277 and PcG proteins are regulated by oxidative stress

**Figure 3:**
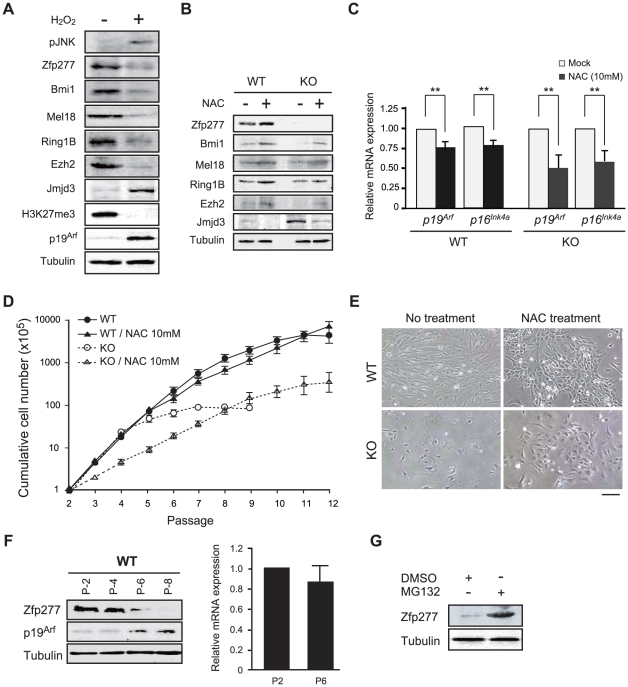
Oxidative stress leads to down-regulation of Zfp277. **A.** H_2_O_2_ treatment of MEFs. The whole-cell extract from wild-type MEFs treated with 100 mM H_2_O_2_ for 6 hrs was subjected to a Western blot analysis for the proteins indicated on the left. **B.** N-acetyl cysteine (NAC) treatment of MEFs. Wild-type and *Zfp277* ^−/−^ MEFs (P-2) were cultured in the presence of 10 mM NAC. MEFs were harvested at passage 6 and the levels of Zfp277 and PcG proteins were determined by Western blotting. Tubulin was used as a loading control. **C.** Quantitative RT-PCR analysis of *p19^Arf^* and *p16^Ink4a^* mRNA in wild-type and *Zfp277* ^−/−^ MEFs at passage 6 cultured in the presence of 10 mM NAC. mRNA levels were normalized to *Hprt1* expression. Expression levels relative to those in wild-type MEFs without NAC treatment are shown as the mean ± SD for three independent experiments. **D.** NAC treatment rescues the growth of *Zfp277* ^−/−^ MEFs. Wild-type and *Zfp277* ^−/−^ MEFs were cultured with or without 10 µM NAC and cell growth was monitored every three days by replating at 1×10^5^ cells/plate. Cumulative cell numbers are shown as the mean ± SD for three independent triplicate experiments. **E.** Photomicrographs of wild-type or *Zfp277* ^−/−^ MEFs with or without NAC observed under an inverted microscope. Scale bar represents 200 µm. **F.** Expression of p19^Arf^ in *Zfp277* ^−/−^ MEFs. The protein levels of p19^Arf^ in wild-type MEFs at the indicated passages were determined by Western blot analyses (left panel). Tubulin was used as a loading control. Quantitative RT-PCR analysis of *p19^Arf^* mRNA in wild-type MEFs at passages 2 and 6 (right panel). mRNA levels were normalized to *Hprt1* expression. Relative expression levels are shown as the mean ± SD for three independent experiments. **G.** MG132 treatment of MEFs. MEFs at passage 4 were treated with 10 µM MG132 for 6 hrs and Zfp277 protein levels were determined by Western blotting. Tubulin was used as a loading control. Statistical significance was determined with Student's t-test; **p<0.01.

In general, culture-induced stress promotes the generation of reactive oxygen species (ROS), the main causative factor for oxidative stress. The balance between the generation and scavenging of ROS is critical for cellular senescence and organismal aging [10], [34]. To assess whether oxidative stress affects the levels of Zfp277 and PcG proteins, we treated MEFs at an early passage (P-2) with hydrogen peroxide (H_2_O_2_) which induces the generation of ROS [35]. At 6 hrs after the treatment with H_2_O_2_ (100 µM), phosphorylation of Jnk, an indicator of activated oxidative stress pathways, was detected (Figure 3A). This treatment led to a significant reduction in Zfp277 and PcG protein (Bmi1, Mel18, Ring1B and Ezh2) levels. The level of H3K27 trimethylation also decreased. In contrast, expression of a histone H3K27 demethylase Jmjd3 was up-regulated (Figure 3A), consistent with previous results showing Jmjd3 expression to be induced in response to stress and oncogenic signaling [31]. Because *Zfp277* ^−/−^ MEFs undergo premature senescence, we next checked their levels of PcG proteins. As demonstrated in Figure 3B, they were decreased in *Zfp277* ^−/−^ MEFs compared to the passage-matched wild-type MEFs. As expected, the level of reactive oxygen species (ROS) was significantly higher in *Zfp277* ^−/−^ MEFs than the wild-type MEFs at the same passage (Figure S3), suggesting that the loss of Zfp277 leads to enhanced oxidative stress which could promote senescence.

An antioxidant, N-acetyl cysteine (NAC), counteracts oxidative stress and extends the replicative life span of primary fibroblasts [36]. NAC treatment increased the protein levels of Zfp277 as well as Bmi1, Ring1B, Mel18, and Ezh2 in wild-type MEFs (P-6) and of Bmi1 and Ezh2 even in *Zfp277* ^−/−^ MEFs (P-6) (Figure 3B). Furthermore, NAC treatment of wild-type and *Zfp277* ^−/−^ MEFs significantly repressed the expression of *p16^Ink4a^* and *p19^Arf^* (Figure 3C) and extended the cells' life span, although NAC itself had a mild adverse effect on proliferation as evident from the growth retardation of *Zfp277* ^−/−^ MEFs at early passages (Figure 3D). At passage 8, *Zfp277* ^−/−^ MEFs stopped growing and exhibited a flattened and enlarged appearance, while those treated with NAC kept growing and maintained a spindle-like shape (Figure 3E). These findings suggest Zfp277 as well as PcG proteins to be regulated by culture-associated oxidative stress in MEFs.

Levels of Zfp277 protein progressively declined during serial passaging, while the mRNA levels of *Zfp277* did not show any significant changes between MEFs at passages 2 and 6 (Figure 3F). Treatment of MEFs with the proteasome inhibitor MG132 remarkably restored levels of Zfp277 protein (Figure 3G). These findings indicate that the Zfp277 level is regulated by protein degradation in a proteasome-dependent manner.

### Antioxidant treatment of *Zfp277* ^−/−^ MEFs restores the binding of PRC2 but not of PRC1 to the *Ink4a/Arf* locus

**Figure 4:**
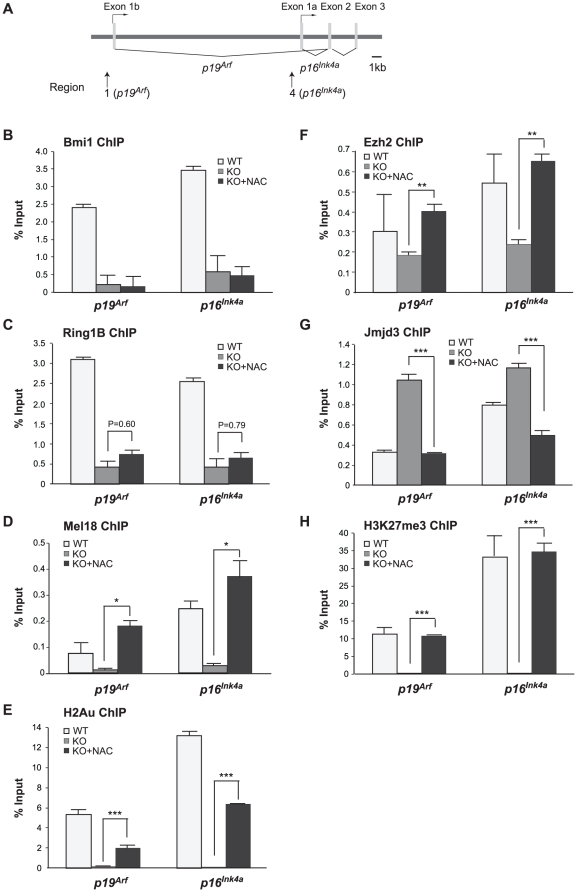
Zfp277 is required for the re-localization of PRC1, but not PRC2. **A.** A schematic representation of the *Ink4a/Arf* locus in MEFs. ChIP assays were performed using anti-Bmi1, Ring1B, Mel18, H2AK119ub1, Ezh2, Jmjd3, and H3K27me3 antibodies. Regions amplified from the precipitated DNA by site-specific quantitative PCR are indicated by arrows. **B–H.** Q-ChIP analysis of the *p19^Arf^* exon 1b and *p16^Ink4a^* exon 1a. Wild-type MEFs and *Zfp277* ^−/−^ MEFs at passage 2 with or without NAC treatment were subjected to ChIP analyses using the indicated antibodies. Percentages of input DNA are shown as the mean ± S.D. for multiple experiments (n>6). Statistical significance was determined with Student's t-test; **p<0.01, ***p<0.001.

Antioxidant treatment downregulated the expression of *p19^Arf^* in *Zfp277* ^−/−^ MEFs and extended the cells' life span during culture *ex vivo*. Therefore, we next tested whether antioxidant treatment can restore the binding of PcG proteins to the *Ink4a/Arf* locus in the absence of *Zfp277*. *Zfp277* ^−*/*−^ MEFs at very early passages (P-2) were treated with NAC and subjected to ChIP assays (Figure 4). NAC treatment successfully restored the binding of the PRC2 core protein Ezh2 and the amount of H3K27me3 to levels comparable to those in wild-type MEFs (P-1) (Figure 4F and H), while reducing the binding of Jmjd3 (Figure 4G). In striking contrast, the binding of Bmi1 was not restored at all and that of Ring1B was only mildly affected (Figure 4B and C). These findings clearly demonstrate that Zfp277 is required for PRC1 to localize to and repress the *Ink4a/Arf* locus and suggest that impaired binding of PRC1 to the *Ink4a/Arf* locus is the primary cause of the derepression which subsequently induces premature senescence in *Zfp277* ^−*/*−^ MEFs. Importantly, however, levels of H2A monoubiquitination moderately but significantly recovered in NAC-treated *Zfp277* ^−*/*−^ MEFs (Figure 4E) and the binding of Mel18 was restored to a level comparable to that in wild-type MEFs (P-1) (Figure 4D). Mel18 is a component of a PRC1-like complex capable of H2AK119 monoubiquitination and can functionally substitute for Bmi1 in many situations [37]. Thus, Zfp277 appears to crosstalk with Bmi1-containing PRC1 but not with the PRC1-like complex containing Mel18.

### Forced expression of Bmi1 cannot bypass premature senescence of *Zfp277* ^−/−^ MEFs

**Figure 5:**
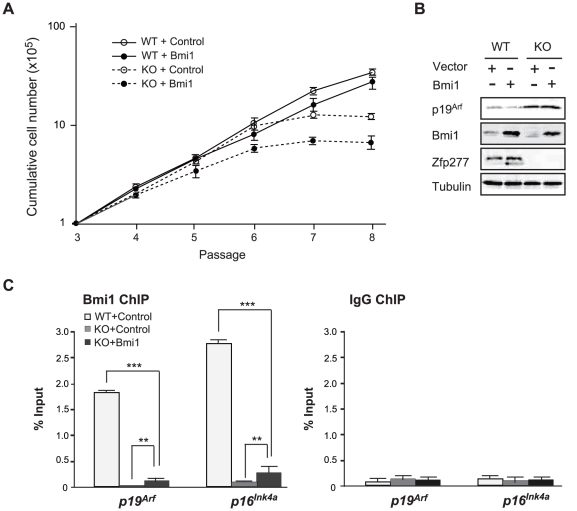
Forced expression of Bmi1 does not repress the *Ink4a-Arf* genes in *Zfp277* ^−/−^ MEFs. **A.** Bmi1 does not rescue *Zfp277* ^−/−^ MEFs from premature senescence. Wild-type and *Zfp277* ^−/−^ MEFs were transduced with either a control or a *Bmi1* retrovirus and cell growth was monitored every three days by replating at 1×10^5^ cells/plate. Cumulative cell numbers are shown as the mean ± SD for three independent triplicate experiments. **B.** Bmi1 does not repress the *Ink4a-Arf* genes in *Zfp277* ^−/−^ MEFs. Wild-type and *Zfp277* ^−/−^ MEFs were transduced with either a control or a *Bmi1* retrovirus and the expression of p19^Arf^ was detected by Western blot analysis. Tubulin was used as a loading control. **C.** ChIP analysis of Bmi1 in Bmi1-transduced *Zfp277* ^−/−^ MEFs. *Zfp277* ^−/−^ MEFs transduced with either a control or a *Bmi1* retrovirus were subjected to ChIP analyses using an anti-Bmi1 antibody and control IgG. The ChIP analysis of Bmi1 in wild-type MEFs at passage 2 is shown as a control. Percentages of input DNA are shown as the mean ± S.D. for three independent experiments. Statistical significance was determined with Student's t-test; **p<0.01, ***p<0.001.

Forced expression of Bmi1 in MEFs extends their life span through tight gene silencing of the *Ink4a/Arf* locus [16], [38]. Therefore, we asked whether Bmi1 could extend the life span of *Zfp277* ^−*/*−^ MEFs. Forced expression of Bmi1 did not abrogate the premature senescence of *Zfp277* ^−*/*−^ MEFs at early passages (Figure 5A). Western blot analysis revealed that exogenous Bmi1 failed to repress the expression of p19^Arf^ (Figure 5B). Furthermore, ChIP analysis of Bmi1 demonstrated that the forced expression of Bmi1 only minimally promoted the binding of Bmi1 to the *Ink4a/Arf* locus in *Zfp277* ^−*/*−^ MEFs (P-2) (Figure 5C). These findings suggest that Zfp277 is required for recruitment of Bmi1 to or its stable accumulation at the *Ink4a/Arf locus* in MEFs.

### Loss of Bmi1 does not affect the distribution of Zfp277 at the *Ink4a/Arf* locus

**Figure 6:**
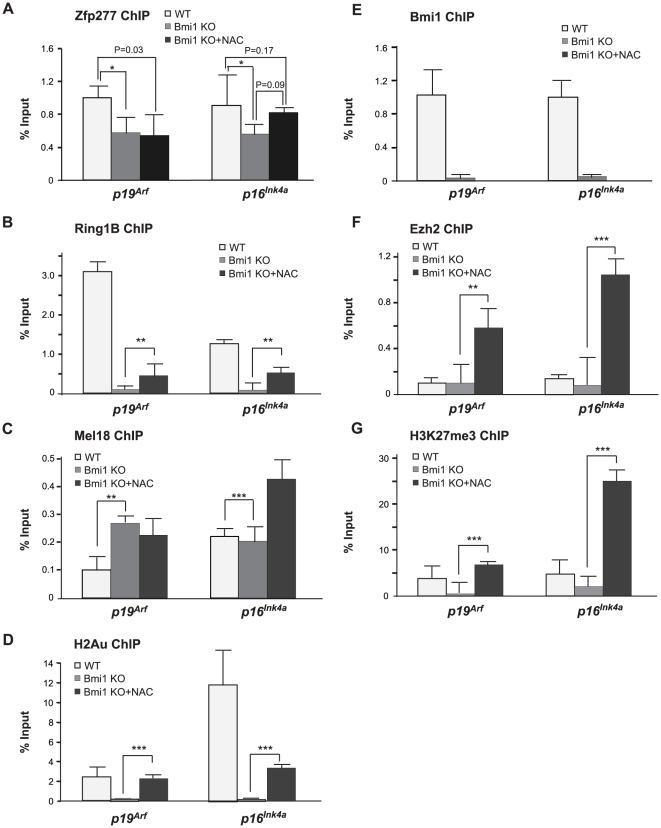
*Bmi1*-deficiency does not greatly affect the binding of Zfp277 to the *Ink4a/Arf* locus in early passage presenescent cells. **A–G.** The ChIP analysis of the *p19^Arf^* exon 1b and *p16^Ink4a^* exon 1a in *Bmi1* ^−*/*−^ MEFs. Wild-type and *Bmi1* ^−*/*−^ MEFs (P-2) pretreated with 10 mM NAC were subjected to ChIP analyses using the indicated antibodies. Percentages of input DNA are shown as the mean ± S.D. for multiple experiments (n>5). Statistical significance was determined with Student's t-test; *p<0.05, **p<0.01, ***p<0.001.

We next asked whether Bmi1 is required for the binding of Zfp277 to the *Ink4a/Arf* locus. *Bmi1* ^−/−^ MEFs undergo premature senescence (<P-6) [15] (Figure S4), and the generation of ROS is reportedly enhanced in the absence of Bmi1 [39]. So, we performed ChIP assays on *Bmi1* ^−/−^ MEFs cultured with or without NAC. ChIP analyses demonstrated that *Bmi1*-deficiency did not greatly affect the binding of Zfp277 to the *Ink4a/Arf* locus in early passage *Bmi1* ^−/−^ MEFs (Figure 6A). NAC treatment had a minimal effect on its binding. Binding of Ezh2 was not grossly altered, either (Figure 6F). In contrast, the binding of the PRC1 protein Ring1B and levels of histone modifications, H3K27me3 and H2AK119ub1, showed a significant reduction in the absence of Bmi1 (Figure 6B, D and G). Interestingly, the binding of Mel18 did not change, or actually enhanced, levels, suggesting a compensatory function (Figure 6C). Again, antioxidant treatment restored or enhanced the binding of Ezh2 and amount of H3K27me3 at the *Ink4a/Arf* locus (Figure 6F and G). In contrast, the binding of Ring1B was restored only partially (Figure 6B). These findings imply that Zfp277 functions as a platform for the Bmi1-containing PCR1 complex but not the Mel18-containing PRC1-like complex at the *Ink4a/Arf* locus.

### Interaction of Zfp277 with Bmi1 is essential to maintain the life span of MEFs

**Figure 7:**
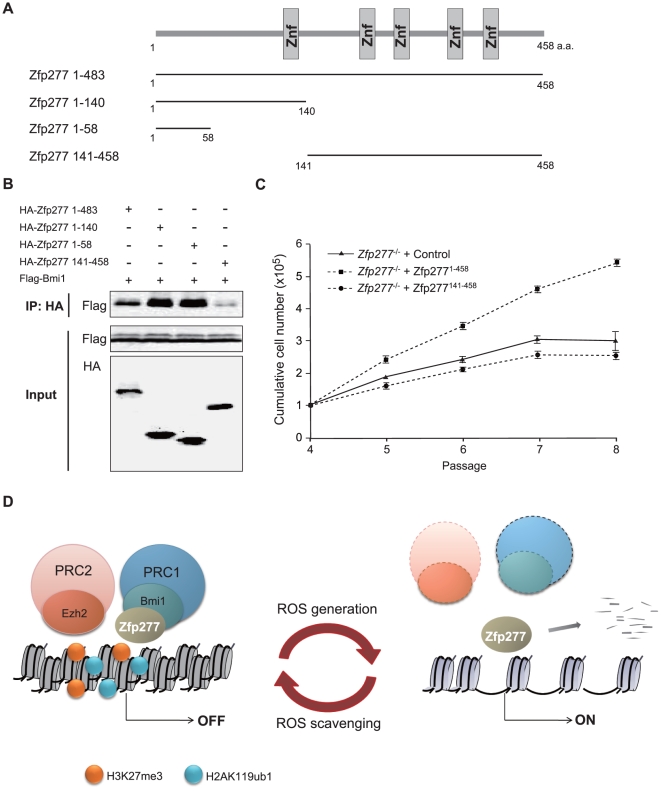
Zfp277 suppresses the *Ink4a/Arf* locus through interaction with Bmi1. **A.** A schematic representation of the deletion mutants of Zfp277. Znf represents a Zinc finger domain. **B.** HA-tagged deletion mutants of Zfp277 and Flag-tagged Bmi1 were cotransfected into 293T cells. After 48 hrs of transfection, the Zfp277 deletion mutants were immunoprecipitated using an anti-HA antibody and the immunoprecipitates were subjected to Western blotting with an anti-Flag or HA antibody. **C.** The growth curve of *Zfp277* ^−/−^ MEFs expressing Zfp277 deletion mutants. *Zfp277* ^−/−^ MEFs (P-3) were transduced with a control, *Zfp277^1–458^*, or *Zfp277^141-458^* retrovirus and cell growth was monitored every three days by replating at 1×10^5^ cells/plate. Cumulative cell numbers are shown as the mean ± SD for three independent triplicate experiments. **D.** A scheme for the dynamic regulation of PcG function in response to oxidative stress. The generation or scavenging of ROS leads to eviction or restoration of Zfp277 and PcG at the *Ink4a/Arf* locus. In this cycle, Zfp277 plays an essential role in the re-localization of PRC1 but not PRC2 in response to the scavenging of ROS.

To further confirm the requirement of physical interaction between Zfp277 and Bmi1 in culture-induced stress-resistance, we first determined the site where Zfp277 interacts with Bmi1 by co-immunoprecipitation using HA-tagged deletion mutants of Zfp277 and a Flag-tagged Bmi1 in 293T cells (Figure 7A). The N-terminal fragments (Zfp277^1–58^, Zfp277^1–140^) retained the capacity to bind to Bmi1, while the C-terminal fragment which lacked the N terminus (Zfp277^141–458^) showed a significant reduction in capacity to bind Bmi1, indicating that the N-terminal portion, Zfp277^1–58^, is the minimal region for interaction with Bmi1 *in vivo* (Figure 7B). We next introduced Zfp277 deletion mutants into *Zfp277* ^−/−^ MEFs and monitored cell proliferation. The expression of wild-type Zfp277 extended the replicative life span of *Zfp277* ^−/−^ MEFs, whereas the expression of Zfp277^141–458^ did not (Figure 7C), despite that the mutant Zfp277 protein was expressed to the same levels of wild-type Zfp277 and retained a nuclear localization (data not shown). These findings underscored the crucial role of the interaction of Zfp277 with Bmi1 in the maintenance of the life span of MEFs.

## Discussion

Our study demonstrated that Zfp277 is critical for the recruitment and/or stable accumulation of Bmi1-containing PRC1 at the *Ink4a/Arf* locus. The localization of Zfp277 was not dependent on the presence of specific PcG histone modifications, H3K27me3 and H2AK119ub1, or the PRC1 proteins Bmi1 and Ring1B. In contrast, neither the overexpression of Bmi1 nor expression of a Zfp277 deletion mutant which cannot bind to Bmi1 prevented the premature senescence of *Zfp277* ^−/−^ MEFs. These findings define Zfp277 as a platform for PRC1 and provide a novel mechanism for PRC1 to lodge at its target loci.

Studies in *Drosophila* have led to the identification of DNA regulatory elements that recruit PcG factors to chromatin, the so-called Polycomb response elements (PREs)(reviewed in Refs [11], [21], [40]). Pleiohomeotic-repressive complex (Pho-RC), which contains a zinc finger DNA-binding protein, Pleiohomeotic (Pho) or Pleiohomeotic-like (Phol), and Scm-like with four MBT domain-containing protein 1 (Sfmbt), directly binds to PREs and recruits PRC2 to PREs. While the study of mammalian PREs is in its infancy, two mammalian PREs, PRE-*kr* and D11.12 elements, have recently been identified, indicating conservation in the mechanisms that target PcG function in mammals and flies [41], [42]. However, a global picture of PRE function in mammals has yet to emerge, and none of the recently characterized PREs have been shown to play roles in later cell survival or senescence. In mammals, PRC2 is recruited to PREs or target gene promoters by the sequence-specific DNA-binding protein yin and yang1 (YY1), the homologue of Pho in *Drosophila* [11], [21], [42]. However, it is unlikely that YY1 is generally required for the recruitment of PRC2 [42]. Instead, several transcription factors (TFs), such as Oct4 in ES cells, have been implicated as recruiters [11]. Furthermore, non-coding RNAs (ncRNAs) and Jarid2/Jumonji, a jumonji C family protein, have been proposed to interact with and recruit PRC2 to target genes [11], [43]. Regarding the recruitment of PRC1, H3K27me3 induced by PRC2 serves as a binding site for CBX, one component of PRC1 [22]. Several CBX family proteins (CBX2, CBX4, CBX7, and CBX8) that have a highly conserved chromodomain can bind to H3K27me3 and participate in the PRC1-mediated transcriptional repression [44], [45]. Nonetheless, it remains controversial whether CBX proteins target PRC1 directly to H3K27me3-marked chromatin [46], [47]. Furthermore, accumulating evidence also suggests that PRC1 recruitment is not completely dependent on H3K27me3 [48]. In this regard, the precise mechanisms for the recruitment of PRC1 remain obscure. Based on our findings, we propose that Zfp277 participates in the recruitment of PRC1 to its target genes and the binding of CBX to H3K27me3 stabilizes the binding of PRC1 to chromatin. Whether this is generally true at all PcG target loci or only applicable to specific loci is an intriguing question to be answered.

Zfp277 contains five repeats of a C_2_H_2_-type zinc finger motif which is found in various transcription factors and capable of binding to specific DNA elements. Among zinc finger proteins, a POZ domain-containing DNA-binding protein, Plzf, is known to interact with Bmi1 and recruit it to target genes [49]. However, we do not know whether the zinc fingers of Zfp277 recognize any specific DNA sequences. It is well established that there is a cohort of zinc finger proteins with highly homologous structures. Therefore, Zfp277 could belong to a family of proteins with overlapping functions. Although the biological impact of *Zfp277*-deficiency awaits detailed analyses of *Zfp277* ^−/−^ mice, the fact that these mice are born healthy raises the possibility that the functions of Zfp277 can be compensated for by other family members *in vivo*.

Cellular senescence is a fail-safe mechanism protecting against genomic instability and tumorigenesis that can be provoked by intra- or extracellular stress. This cell-autonomous tumor barrier is often attributed to deregulation of the *Ink4a/Arf* locus. We have demonstrated that levels of Zfp277 protein are reduced in response to culture-induced oxidative stress through a proteasome-dependent pathway of degradation in wild-type MEFs. Furthermore, Zfp277 is immediately degraded when MEFs are exposed to UV and X-ray irradiation (data not shown), indicating that it is targeted by a broad range of stress-related pathways. Similarly, the accumulation of ROS triggers a reduction in PcG protein levels downstream of activated JNK signaling [50], [51]. We also observed that levels of ROS in MEFs inversely correlated with levels of Zfp277 and PcG proteins. The fact that both Zfp277 and PcGs are crucial for repression of the *Ink4a/Arf* locus and inversely regulated downstream of stress pathways further supports that functional crosstalk occurs between Zfp277 and PcGs. Together, our findings indicate that PcG function is dynamically regulated in response to oxidative stress. The generation or scavenging of ROS leads to the eviction or restoration of Zfp277 and PcG proteins at the *Ink4a/Arf* locus. In this cycle, Zfp277 plays an essential role in the re-localization of PRC1 but not PRC2 (Figure 7D).

NAC treatment of both *Zfp277* ^−/−^ and *Bmi1* ^−/−^ MEFs restored the binding of only PRC2, not PRC1, to the *Ink4a/Arf* locus. Nonetheless, it efficiently achieved a repressive state at the *Ink4a/Arf* locus and overrode the limited replicative life span of MEFs. Of importance, our ChIP analysis showed that neither Zfp277 nor Bmi1-deficiency affected the distribution of Mel18 at the *Ink4a/Arf* locus in the presence of NAC, suggesting that Mel18 is recruited to the *Ink4a/Arf* locus independently of Zfp277 or Bmi1. It is reported that human MEL18 forms a BMI1-independent PRC1 complex with RNF2, exhibiting E3 ubiquitin ligase activity for histone H2A [37]. Mel18 and Bmi1 knockout mice showed similar phenotypes at least for cell proliferation [15], [52] and the knockout of both genes revealed that they synergistically maintain Hox gene expression during development [53]. Furthermore, *Mel18* ^−/−^ MEFs undergo p19^Arf^-dependent premature senescence like *Bmi1* ^−/−^ MEFs [54]. Our findings indicate that Mel18 behaves as a back-up molecule for Bmi1 in gene silencing of *Ink4a/Arf* in *Zfp277* ^−/−^ and *Bmi1* ^−/−^ MEFs treated with NAC, thus supporting the notion of functional redundancy between Bmi1 and Mel18. However, the specific collaboration between Zfp277 and Bmi1-containing PRC1 also highlights the difference in properties between Bmi1- and Mel18-containing PRC1.

Although Zfp277 is not responsible for the recruitment of Ezh2 and Mel18, the binding of these two proteins to the *Ink4a/Arf* locus was profoundly affected in *Zfp277* ^−/−^ MEFs (Figure 4). This might be attributed to enhanced oxidative stress, as evident in Figure S3, which causes a reduction in the levels of Ezh2 and Mel18 (Figure 3A). This notion is supported by the results of ChIP experiments with NAC-treated *Zfp277* ^−/−^ MEFs in which the recovery in binding of Ezh2 and Mel18 to the *Ink4a/Arf* locus was well correlated with the recovery of their protein levels. In the same experiments, the H3K27me3 levels were reduced to background levels, although the reduction in Ezh2 binding was not so drastic in *Zfp277* ^−/−^ MEFs (Figure 4). This discrepancy could be explained by the enhanced recruitment of Jmjd3, an H3K27me2/3 demethylase, to the *Ink4a/Arf* locus. Furthermore, it is hypothesized that there is an interdependence of PRC1 and PRC2 binding and function [41]. These factors could account for a significant reduction in H3K27me3 at the *Ink4a-Arf* locus in *Zfp277* ^−/−^ MEFs.

Expression of p16^Ink4a^ increases markedly in aging mouse and human tissues, linking *Ink4a/Arf* with the aging process [55]. Importantly, numerous studies have shown that aging is closely associated with oxidative damage. For example, the generation of ROS leads to a cell-autonomous functional decline of hematopoietic stem cells which normally exhibit low levels of ROS. Conversely, NAC treatment prevents the stress-induced loss of hematopoietic stem cells [56]. In this study, we observed that ROS profoundly affected the functions of both Zfp277 and PcG proteins and induced JNK activation, resulting in deregulation of the *Ink4a/Arf* locus. Thus, our results raise the possibility that Zfp277 is involved in the regulation of aging and stem cell function through collaborative regulation of the *Ink4a/Arf* locus with PcG complexes.

## Materials and Methods

### Plasmids and antibodies

Full-length mouse *Zfp277* (NM_178845) was cloned by RT-PCR using mouse hematopoietic progenitor cDNA. HA-tagged *Zfp277* and deletion mutants, Flag-tagged *Bmi1* fused with *GFP,* Flag-tagged *Ring1B*, Flag-tagged *Ezh2*, and Myc-tagged *Mel18* were subcloned into pcDNA3 (Invitrogen). Flag-tagged *Bmi1* fused with *GFP* was also subcloned into CSII-EF-1α lentivirus vector. Full-length *Zfp277* and Zfp277^141-458^ were subcloned into a retroviral vector, MSCV-EF-1-EGFP. The GST fused with Zfp277 or Bmi1 was subcloned into pGEX6p-1 expression vectors (Invitrogen). The retroviral vector encoding Bmi1 was described previously [57]. pMYs-E6-ires-EGFP was kindly provided by Dr. T. Kiyono (National Cancer Center, Tokyo, Japan). A polyclonal antibody against Zfp277 was generated by immunizing rabbits with recombinant N-terminal Zfp277 (a.a. 1–170).

### Generation of Zfp277 knockout mice and preparation of MEFs

Gene targeting of *Zfp277* was performed as described previously [58]. Briefly, a gene-targeting vector was generated using a BAC clone containing the *Zfp277* gene obtained from a mouse 129/Sv BAC library (Invitrogen). The targeting strategy is shown in Figure S1. The linealized targeting vector was introduced into R1 ES cells by electroporation and the cells were subjected to selection using G418. G418-resistant clones were isolated and recombination was confirmed by Southern blotting using an internal probe. Germline chimeras were generated by an aggregation method. Germline transmission of the target allele was confirmed by PCR using DNA from the tail. Mice were bred and maintained in the Animal Research Facility of the Graduate School of Medicine, Chiba University in accordance with our institutional guidelines. This study was approved by the institutional review committees of Chiba University (approval numbers 21–65 and 21–150). MEFs prepared from E12.5 embryos were cultured in Dulbecco's modified Eagle's medium (DMEM) supplemented with 10% fetal bovine serum and a 1% penicillin-streptomycin mixture (Sigma) at 37°C under 10% CO2 in a humidified incubator and passaged every three days.

### Cell proliferation assay, β-galactosidase staining, and antioxidant treatment

For the cell proliferation assay, wild-type, *Zfp277* ^−/−^, and *Bmi1* ^−/−^ MEFs were seeded at 1×10^5^ cells/well in 6-cm plates and cultured at 37°C in 10% CO2 in a humidified incubator. Viable cells were counted every three days by Trypan Blue exclusion, and then replated at 1×10^5^ cells/well. Antioxidant treatment was performed by adding 10 mM NAC (Sigma) to the medium. For the staining of β-galactosidase, cells were fixed with 3% formaldehyde and 0.025% glutaraldehyde (Sigma) for 5 min, and incubated with citrate buffer (pH 5.2) containing FeCN for 2 to 16 hrs before images were taken.

### RNA extraction, reverse transcription and quantitative PCR analysis

Total RNA was extracted from cells at 75% confluency using Trizol LS^®^ total RNA isolation reagent (Gibco BRL, Life Technologies) according to the manufacturer's instructions. cDNA was synthesized from total RNA using the Superscript II First Strand Synthesis System (Invitrogen). Quantitative RT-PCR was performed with an ABI Prism® 7300 Sequence Detector (Applied Biosystems). Universal Library probe assays were performed using the LightCycler® FastStart universal probe master (PE Applied Biosystems), an optimal combination of universal probe, and gene-specific primer sets designed by the Universal ProbeLibrary assay design center (https://www.roche-applied-science.com) (Table S1). The reactions were conducted in a 96-well optical plate at 95°C for 10 min, followed by 40 cycles of 95°C for 5 sec and 60°C for 31 sec. The Ct data was determined with default threshold settings.

### Immunoprecipitation, co-Immunoprecipitation, and Western blot analyses

For immunoprecipitation and co-immunoprecipitation analyses, cells were lysed in hypertonic lysis buffer [20 mM sodium phosphate at pH 7.0, 250 mM NaCl, 0.1% NP-40, 30 mM Na_4_P_2_O_7_, 10 mM NaF, 5 mM EDTA, 1 mM DDT, and a protease inhibitor cocktail (Complete Midi, Roche)] at 4°C for more than 40 min, sonicated, and subjected to immunoprecipitation as described previously [27]. Proteins were analyzed by SDS-PAGE, transferred to a PVDF membrane (Immobilon™; Millipore), and detected by Western blotting using the following antibodies; HA (sc-805, Y-11), and p16^Ink4a^ (sc-468, C-20)(Santa Cruz); Bmi1 (05-637, F6), H3K27me3 (07-449)(Millipore); Kdm6b/Jmjd3 (ab38113), p19^Arf^ (ab80)(Abcam); α-Tubulin (T6074, B-5-1-2)(Sigma); phosphor-Sapk/Jnk (Thr183/Try185)(#9251) (Cellsignal); Ezh2 (BD43) [59], and mouse anti-Ring1B monoclonal antibody [60].

### Chromatin immunoprecipitation assay

ChIP assays were performed as described previously [27]. Briefly, cells were fixed with 1% formaldehyde for 15 min and then incubated in the presence of 0.125 M Glycine for 10 min. The cells were lysed with lysis buffer [(50 mM Tris-HCl (pH 8.0), 10 mM EDTA, 0.1%SDS, and a proteinase inhibitor cocktail (Complete Midi)) on ice for 20 min and sonicated. The lysate was separated into three samples diluted with an equal volume of dilution buffer [50 mM Tris-HCl (pH 8.0), 150 mM NaCl, 1%Triton X-100, 0.1%SDS, and a proteinase inhibitor cocktail (Complete Midi)]. Immunoprecipitation was performed overnight at 4°C using anti-Bmi1 (clone 8A9, kindly provided by Dr. N. Nozaki, MAB Institute, Co. Ltd., Japan), anti-monoubiquitinated H2A (H2Aub1) (E6C5, Millipore), anti-rabbit IgG, anti-mouse IgG, anti-Ring1B, anti-Kdm6b/Jmjd3, anti-Mel18 (sc-10744, H-115)(SantaCruz), anti-Ezh2 (AC22)[55], anti-HA, anti-H3K27me3, and anti-Zfp277 antibodies. For anti-H2Aub1, chromatin was immunoprecipitated overnight at 4°C with anti-H2Aub1, followed by the addition of anti-mouse IgMµ (12-488, Millipore) and further incubation for 2 hours at 4°C. After the immunoprecipitation, 25 ml of salmon sperm DNA/Protein G and A sepharose beads was added and incubated for 1 hr at 4°C. The immunoprecipitates were washed extensively and subjected to a Q-PCR analysis by SYBR Premix Ex Taq™ II (Takara) using the primers listed in Supplemental Table S1 [33].

## Supporting Information

Figure S1 (2.29 MB EPS)

Figure S2 (1.74 MB EPS)

Figure S3 (1.65 MB EPS)

Figure S4 (1.38 MB EPS)

Table S1 (1.50 MB EPS)
